# Current Directions in the Auricular Vagus Nerve Stimulation I – A Physiological Perspective

**DOI:** 10.3389/fnins.2019.00854

**Published:** 2019-08-09

**Authors:** Eugenijus Kaniusas, Stefan Kampusch, Marc Tittgemeyer, Fivos Panetsos, Raquel Fernandez Gines, Michele Papa, Attila Kiss, Bruno Podesser, Antonino Mario Cassara, Emmeric Tanghe, Amine Mohammed Samoudi, Thomas Tarnaud, Wout Joseph, Vaidotas Marozas, Arunas Lukosevicius, Niko Ištuk, Antonio Šarolić, Sarah Lechner, Wlodzimierz Klonowski, Giedrius Varoneckas, Jozsef Constantin Széles

**Affiliations:** ^1^Institute of Electrodynamics, Microwave and Circuit Engineering, Vienna University of Technology, Vienna, Austria; ^2^SzeleSTIM GmbH, Vienna, Austria; ^3^Max Planck Institute for Metabolism Research, Cologne, Germany; ^4^Cologne Cluster of Excellence in Cellular Stress and Aging Associated Disease (CECAD), Cologne, Germany; ^5^Neurocomputing and Neurorobotics Research Group, Complutense University of Madrid, Madrid, Spain; ^6^Laboratory of Neuronal Networks, Department of Mental and Physical Health and Preventive Medicine, University of Campania “Luigi Vanvitelli”, Naples, Italy; ^7^Ludwig Boltzmann Cluster for Cardiovascular Research at the Center for Biomedical Research, Medical University of Vienna, Vienna, Austria; ^8^Foundation for Research on Information Technologies in Society, Zurich, Switzerland; ^9^Department of Information Technology, Ghent University/IMEC, Ghent, Belgium; ^10^Biomedical Engineering Institute, Kaunas University of Technology, Kaunas, Lithuania; ^11^Faculty of Electrical Engineering, Mechanical Engineering and Naval Architecture, University of Split, Split, Croatia; ^12^Nalecz Institute of Biocybernetics and Biomedical Engineering, Polish Academy of Sciences, Warsaw, Poland; ^13^Sleep Medicine Centre, Klaipeda University Hospital, Klaipëda, Lithuania; ^14^Institute of Neuroscience, Lithuanian University of Health Sciences, Palanga, Lithuania; ^15^Department for Surgery, Medical University of Vienna, Vienna, Austria

**Keywords:** auricular vagus nerve, nerve stimulation, biophysics, brain plasticity, inflammation, animal research, clinical studies

## Abstract

Electrical stimulation of the auricular vagus nerve (aVNS) is an emerging technology in the field of bioelectronic medicine with applications in therapy. Modulation of the afferent vagus nerve affects a large number of physiological processes and bodily states associated with information transfer between the brain and body. These include disease mitigating effects and sustainable therapeutic applications ranging from chronic pain diseases, neurodegenerative and metabolic ailments to inflammatory and cardiovascular diseases. Given the current evidence from experimental research in animal and clinical studies we discuss basic aVNS mechanisms and their potential clinical effects. Collectively, we provide a focused review on the physiological role of the vagus nerve and formulate a biology-driven rationale for aVNS. For the first time, two international workshops on aVNS have been held in Warsaw and Vienna in 2017 within the framework of EU COST Action “European network for innovative uses of EMFs in biomedical applications (BM1309).” Both workshops focused critically on the driving physiological mechanisms of aVNS, its experimental and clinical studies in animals and humans, *in silico* aVNS studies, technological advancements, and regulatory barriers. The results of the workshops are covered in two reviews, covering physiological and engineering aspects. The present review summarizes on physiological aspects – a discussion of engineering aspects is provided by our accompanying article (Kaniusas et al., 2019). Both reviews build a reasonable bridge from the rationale of aVNS as a therapeutic tool to current research lines, all of them being highly relevant for the promising aVNS technology to reach the patient.

## Introduction

Bioelectronic medicine progressively comes into focus as a non-pharmaceutical treatment option for various diseases. Here neuromodulation of the vagus nerve (VN), known also as wandering or pneumogastric nerve, gained a special interest in recent years.

This review aims to summarize the contemporary views on the electrical stimulation of the auricular VN (aVNS) as a promising electroceutical therapy in humans. Catalysts were the first two international workshops on aVNS in Warsaw (February 16, 2017) and Vienna (October 26 and 27, 2017) within the scope of EU COST Action “European network for innovative uses of EMFs in biomedical applications (BM1309).” In particular, the present review summarizes and discusses the physiological role of VN including a biology-driven rationale for aVNS, backed up by experimental and clinical data. A focused review on technical issues, modeling concepts, regulatory requirements, and novel architectures of aVNS paradigms is provided in our accompanying article (Kaniusas et al., 2019).

We start with biophysical principles underlying aVNS and continue with modulation of different body functions while including experimental data in animals and clinical data in humans. Future directions of aVNS are identified to complement this review.

### Vagus Nerve

The vagus nerve is the 10th cranial nerve that starts at the brainstem with two bilateral branches, and widely meanders and loops within the neck, thorax and abdomen (Trepel, 2017). VN is composed out of myelinated A and B fibers as well as non-myelinated C fibers. VN establishes a mutual connection between the brain and major body structures as pharynx, larynx, trachea, heart, aorta, lungs, and the entire gastrointestinal tract including esophagus, stomach, liver, pancreas, and spleen (Berthoud and Neuhuber, 2000; Standring, 2016). These widespread projections of VN imply its involvement in many functions of the body’s autonomic nervous system (ANS). The activity of VN is proportionally associated with health, wellbeing, relaxation, and even emotions like empathy, whereas it is negatively associated with risk factors such as morbidity, mortality, and stress (Thayer et al., 2009; Zulfiqar et al., 2010). VN thus plays a crucial role in determining brain-body interactions (de Lartigue, 2016). These manifold interactions – as best exaggerated in Clancy et al. (2012) as “My function’s almost anything, and vagus is my name” – naturally cause increasing interest in artificial VN stimulation for therapeutic purposes. VN is considered as a major nerve and thus mediator of the parasympathetic section of ANS, whereas the vagal tone activates the parasympathetic nervous system (Olshansky et al., 2008; Barella et al., 2014).

**FIGURE 1:**
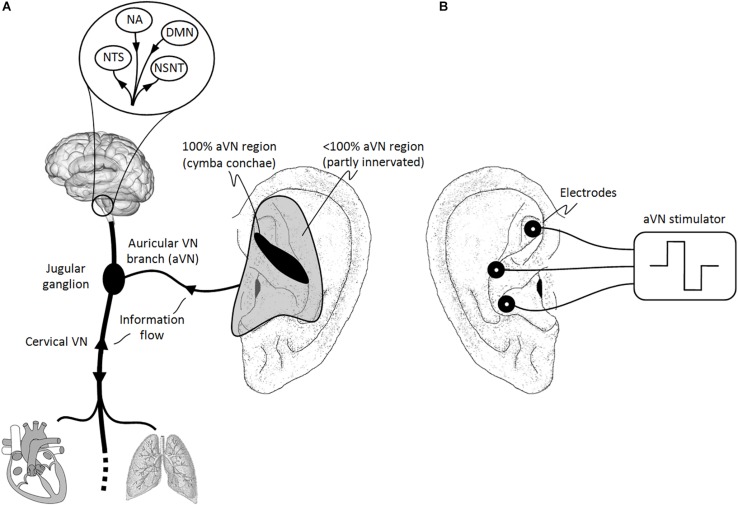
Natural sensory innervation of the auricle versus its artificial stimulation. **(A)** The vagus nerve (VN) connects the brain with most of the organs within the thorax and abdomen. Afferent auricular branches (aVN) leave the cervical VN at the level of the jugular ganglion just outside the cranium and innervate the rather central regions of the pinna of the outer ear (Peuker and Filler, 2002). **(B)** Electric stimulation of aVN endings with needle electrodes located within these central regions. NTS, nucleus of the solitary tract; NSNT, nucleus spinalis of the trigeminal nerve; NA, nucleus ambiguous; DMN, dorsal motor nucleus. This figure and figure caption was originally published in the sister manuscript to this review (Kaniusas et al., 2019), which was published in Frontiers of Neuroscience under the creative commons attribution license CC BY 4.0.

Most VN fibers (about 80%) are afferent sensory fibers carrying somatic and visceral information to the brainstem and thus providing a unique entrance to the brain (Berthoud and Neuhuber, 2000; Groves and Brown, 2005). In particular, visceral and cranial homeostatic sensory activity is mediated by input from VN (and other nerves) to the nucleus of the solitary tract (NTS) in the lower medulla. As shown in Figure 1A, most of afferent fibers of VN end in NTS, e.g., for visceral, taste, heart, and aorta afferents, while other afferents terminate in the nucleus spinalis of the trigeminal nerve, e.g., for larynx and pharynx afferents.

The rest of VN fibers (about 20%) are efferent visceromotor fibers governing neurogenic, myogenic, and endocrine actions within end organs. These motor fibers originate in the nucleus ambiguous (for muscle innervation of the pharynx and larynx) and in the dorsal motor nucleus (to supply heart, lungs, esophagus, and stomach), as illustrated in Figure 1A. For instance, the right VN is more closely associated with the cardiac atria and innervates the sinoatrial node controlling the heart rate while the left VN is rather associated with the ventricles of the heart and innervates the atrioventricular node controlling the contraction force (Guiraud et al., 2016).

Considering our focus on vagus nerve stimulation (VNS), VN connects specific sensors and effectors in the periphery with the central nervous system. Mediated connections of VN – within reach by several synaptic connections – include projections to hypothalamus and cortex as higher brain regions, thus allowing modulative access of VN to subcortical and cortical brain areas (Berthoud and Neuhuber, 2000). Therefore, signals generated in VN have the potential to affect a broad range of basic brain functions and thus to affect the entire organism in terms of its protection.

### Sensory Functions

Since VN is mainly a sensory nerve, it essentially relays biofeedback to the brain, which originates in mechanoreceptors, chemoreceptors, thermoreceptors, and osmoreceptors spread throughout the whole body (Berthoud and Neuhuber, 2000; Szekely, 2000; Williams et al., 2016). Most afferent VN endings are polymodal responding to a variety of stimuli. Here a direct activation of free nerve endings of the afferent VN, so-called primary sensing cells with embedded receptors in the nerve membrane (Kaniusas, 2012), are likely to be involved; e.g., for the sense of pain. In addition, an indirect activation of free endings of the afferent VN is given through synaptically connected specialized cells, so-called secondary sensing cells, e.g., for the sense of taste (de Lartigue and Diepenbroek, 2016).

To give a few examples, venous baroreceptors (mechano- receptors) connect to the afferent VN for blood volume control. Arterial baroreceptors in the aortic arch relay blood pressure via the afferent VN to the brainstem and thus serve as feedback for the short-term control of the arterial blood pressure (baroreflex). Mechanoreceptors and chemoreceptors in the stomach detect its luminal contents and signal it via the afferent VN to the brain for the satiety/hunger response (Williams et al., 2016; Andermann and Lowell, 2017). Here the afferent VN fibers originate in the mucosa and muscle layers of the digestive tract. Pulmonary stretch is also conveyed via the afferent VN to the brain, whereas mechanoreceptors and chemoreceptors in the respiratory tract govern the cough reflex. Likewise, mechanical stimulation of the afferent VN receptors in the diaphragm seems to mediate the influence of paced breathing to the brainstem activity. Interestingly, the Valsalva maneuver, vomiting reflex, carotid sinus massage, and ocular compression can be intentionally used to alter VN activity (Clancy et al., 2012). The afferent VN receptors strongly connect enterocrine cells with the brain (Han et al., 2018) and link this axis to reward sensitivity (Kaelberer et al., 2018).

The afferent VN carries also nociceptive fibers for pain, i.e., sensing extremes in temperature, pressure, or chemicals with high-threshold receptors. For instance, tearing or burning sensations related to irritation of lower airways are transmitted mainly through VN afferents. Further, a noxious gastric distention results in the vagally mediated excitation of NTS, whereas cardiac ischemia and pain are also mediated by VN receptors (Berthoud and Neuhuber, 2000).

### Body Control via Sensory Functions

**FIGURE 2:**
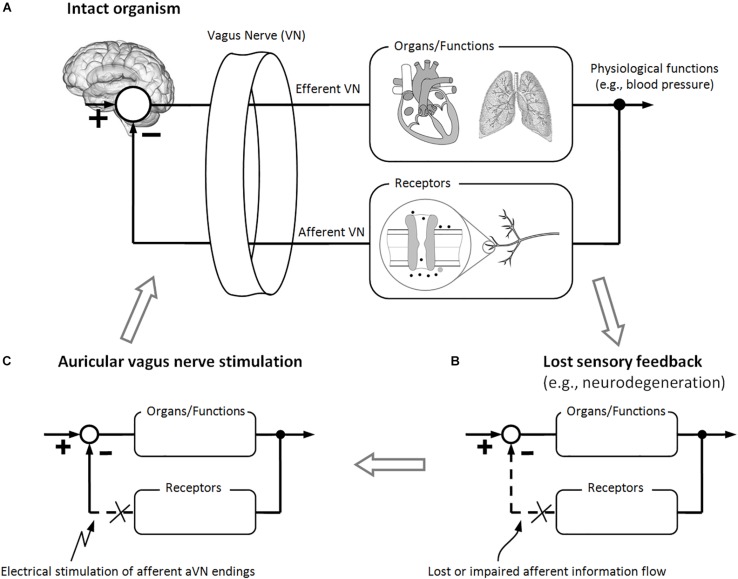
Brain modulation via electrical stimulation of auricular vagus nerve (aVN) endings. **(A)** Intact feedback-loop which is composed out of efferent VN fibers (controlling different organs and functions) and afferent VN fibers (carrying sensory information back to the brain) for proper control of bodily organs and functions. **(B)** Different diseases may lead to a lost or impaired afferent feedback to the brain (e.g., due to neurodegeneration or maladaptive plasticity), which makes it impossible for the brain (the control station of the body) to adapt to changes in organs, functions, and/or environmental factors. **(C)** As a hypothesis, stimulation of aVN fibers substitutes the lost or impaired afferent feedback to the brain while inducing systemic regeneration processes. These processes, in turn, may lead to sustainable recovery of controlled organs and functions as well as recovery of the relevant sensory feedback-loop.

Activation of vagal afferents elicits efferent parasympathetic activation and sympathetic inhibition, like in the baroreflex (Karemaker, 2017). Therefore, a negative inhibitory feedback is provided by VN receptors to otherwise feed-forward sympatho-excitatory activation. As shown in Figure 2A, sensory VN fibers establish feedback-based control loops with their, in general, non-linear dynamics. However, since many organs such as the heart are innervated by both sympathetic and parasympathetic fibers, it is more precise to speak of the parallel control by both systems than to stress the antagonistic actions of parasympathetic and sympathetic systems (Olshansky et al., 2008; Karemaker, 2017).

Sensory fibers in VN provide an excellent opportunity for the body to detect its homeostasis in the periphery (e.g., immune-related events) and then to generate appropriate neurogenic, myogenic, endocrine, and behavioral responses. These responses involve typically closed-loop reflex pathways along efferent and afferent pathways (Figure 2A). Central integration and processing of sensory information and the associated generation of motor output take place in the brain. Prominent examples are thermo-regulation, immune-regulation, and blood pressure control of the body.

For instance, increased arterial blood pressure (hypertension) stimulates baroreceptors in the aortic arch, signaling via the afferent VN to the brainstem. The brain, especially, NTS inhibits reflexively the sympathetic outflow to the heart and periphery in response to hypertension, whereas the stroke volume and the total peripheral resistance decrease, respectively. The parasympathetic outflow via efferent VN fibers is reflexively accelerated, slowing the heartbeat via the efferent VN connection to the sinoatrial node of the heart. Consequently, the blood pressure drops and thus normalizes. One can experience this vagal-mediated decrease in the heart rate through gentle rubbing on the vagus nerve via the carotid sinus, a technique known as carotid massage (Schweitzer and Teichholz, 1985; Lim et al., 1998).

Collectively, sensory VN seems to provide a powerful back door into the body, allowing you to “hack the brain” via artificial stimulation of sensory VN fibers, provided that the electrical language of nerves is spoken (Moore, 2015).

## Why Auricular Vagus Nerve?

The external ear is the only place on the body where VN sends its only peripheral branch. In fact, the auricular branch of VN surfaces as the afferent auricular VN (aVN) and thus forms a cutaneous receptive field in the pinna of the ear. This field is susceptible to external stimuli in terms of peripheral nerve stimulation. In particular, aVN allows for an easy external access via electrical stimulation in terms of aVNS, which then connects directly and favorably the applied stimuli to the brainstem, as shown in Figure 1B. The brainstem even mediates aVNS input to higher brain regions via extensive projections to second and third order neurons within the brain (Mercante et al., 2018b). The auricle and especially its aVN endings might become a powerful direct gateway to modulate various brain functions, offering the most affordable non-invasive manipulation of the central nervous system.

Even at about 100BC, the auricle’s importance for acupuncture therapy was recognized as “All the vessels congregate in the ear” (Sator-Katzenschlager and Michalek-Sauberer, 2007). Current evidence suggests that even antinociceptive effects of auricular acupuncture are mediated by the stimulation of aVN (Usichenko et al., 2017).

Auricular vagus nerve is also known as Arnold’s or Alderman’s nerve (Nomura and Mizuno, 1984). It leaves the cervical VN at the level of the jugular ganglion where aVN have their bodies of the sensory ganglionic neurons, just outside the cranium. aVN endings provide the sensory innervation of specific regions of the external ear (Alvord and Farmer, 1998; Kandel et al., 2000; Peuker and Filler, 2002; He et al., 2012). As illustrated in Figure 1A, the middle region of the pinna, the central concha, is mostly innervated by aVN, whereas aVN was found in 100% of cases in cymba concha (Peuker and Filler, 2002). Other ear regions such as antihelix, cavity of concha, tragus, crus of helix, and crura of antihelix were found to be partly but non-exclusively innervated by aVN in 73, 45, 45, 20, and 9% of cases, respectively (Peuker and Filler, 2002). aVN is composed out of myelinated Aβ fibers (fibers with the diameter 7–10 μm comprise about 20% of the total myelinated aVN axons with about 370 myelinated axons per auricle), myelinated Aδ fibers (fibers with the diameter 2–5 μm comprise about 50% of the total myelinated aVN axons) (Safi et al., 2016), and non-myelinated C fibers (Standring, 2016).

Besides aVN endings, the ear contains endings of non-vagal cervical and cranial nerves such as the great auricular nerve (e.g., present in the ear lobe), the auriculotemporal nerve (e.g., the branch of the trigeminal nerve located in the spine of helix), and the lesser occipital nerve (e.g., the upper third of the medial surface of the auricle) (Peuker and Filler, 2002). From an anatomical point of view, all nerve fibers in the auricle run between the ear cartilage and skin in a depth of 1–1.5 mm (Bermejo et al., 2017). From a functional point of view, different physiological effects can be expected when stimulating locally different areas in the ear. For instance, variable intensity of induced autonomic changes was shown as a function of the area of stimulation in rats (Gao et al., 2007), whereas a significantly stronger activation of the NTS was proven when the cymba concha region was electrically stimulated in humans compared to sham stimulation and other auricular regions (Yakunina et al., 2016).

Auricular vagus nerve stimulation recruits sensory aVN fibers and thus mimics/projects sensory input to the brainstem in terms of neuromodulation, forming the so-called auriculo-vagal afferent pathway (He et al., 2013). Since aVNS projects directly to NTS (Figure 1A), both ANS and the central nervous system are modulated by aVNS. Consequently, since ANS, composed out of sympathetic and parasympathetic branches, governs systemic parameters of cardiovascular, respiratory, and immunological functions to stay within their homeostatic limits and, on the other hand, aVNS modulates the parasympathetic auricular branch, aVNS effects on the body can be expected to be systemic.

Systemic effects affect multiple physiological functions and are not specifically targeted to a particular organ or an isolated function. For instance, the sympathovagal balance – as a systemic parameter of the body – can be modulated for therapeutic reasons. In line with the aforementioned inhibitory effects of vagal receptors, systemic effects of aVNS can expected to be mostly from the sympatho-inhibitory origin (Deuchars et al., 2017). As illustrated in Figure 2A, aVNS modulates the biofeedback line to the brain so that this modulation shapes closed-loop reflex pathways, in analogy with neurobiological models from De Couck et al. (2011). Therefore, once again, diverse systemic effects of aVNS can be expected on the entire organism.

From an evolutionary developmental perspective, aVN is all that remains of a more extensive embryonic nerve which supplies the first branchial arch and is supposed to be the last phylogenetic remnant of the nerve innervating the lateral line organs in fish and amphibia (He et al., 2013). Interestingly, the sensory auricular nerve endings (including aVN) in mammals may have evolved from the mandibular/jaw area in the course of the evolutionary transition when post-dentary jaw elements moved to the cranium as auditory ossicles (Meng et al., 2011). This evolutionary development may justify some current complex pathways of aVN action in the pharynx area and may shed some light on the physiological relevance of aVN endings today.

## Vagus Nerve Stimulation – From Biophysics to Animal and to Human Data

Auricular vagus nerve stimulation is a peripheral, non-pharmacological, and minimally invasive neuromodulation technique, altering signal processing in the central nervous system, activating reflex circuitries, exploiting brain plasticity for different therapeutic purposes, and thus affecting profoundly different areas of the brain, as described below. The broad range of brain versus periphery projections of VN and its corresponding functions suggest a large variety of disorders possibly indicated for aVNS therapy in humans, whereas these disorders share common features related to the protective function of VN. This simple peripheral technique of aVNS gains access to central pathways of the brain. Due to systemic effects of aVNS – and, in general, of any VNS – many different biophysical mechanisms have been found to be modulated, including ANS affecting the whole organism. Though the exact mechanisms of aVNS and VNS remain to be elucidated, the following neurophysiological evidence and modulation of different body functions are based on strong hypothesis derived from experimental animal and clinical human data.

With the critical assumption that observed neurophysiological effects of aVNS and VNS may be considered as being similar (see below), here we review both implanted VNS and non-implanted minimally invasive aVNS in animal models. In fact, tightly controlled settings in experimental animal studies allow for potentially more consistent findings in bio-physiological basic mechanisms behind aVNS than human studies. In addition, observed effects in animals indicate potential applications of aVNS in humans.

**FIGURE 3:**
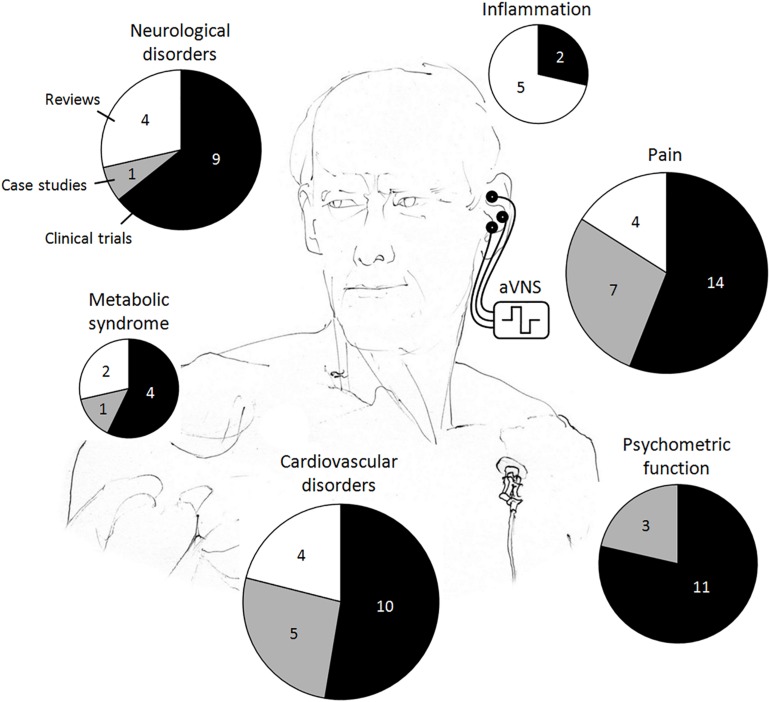
Reported clinical applications of the auricular vagus nerve stimulation (aVNS) in humans. The total area of the pie chart indicates the total number of publications reviewed, whereas individual pieces refer to the respective numbers of clinical trials, case studies, and reviews.

In the reviewed human studies, we focus on clinical evidence of transcutaneous and percutaneous aVNS, whereas a lot of populations have been found to benefit from aVNS therapy, as summarized in Figure 3. Although randomized clinical trials on aVNS are accumulating, a lot of them are preliminary case studies. For human studies with potential clinical applications, we differentiate explicitly between clinical trials, case studies, and reviews to qualify the strength of statement. Studies on auricular electroacupuncture or peripheral nerve stimulation are also included if stimulation electrodes were located in vagally innervated regions of the auricle (Figure 1A). Therefore, applications of the implanted VNS in humans – the therapy which became established during the past 20 years (Beekwilder and Beems, 2010) – are excluded in order to focus on aVNS only.

### Neurophysiological Evidence – Modulation of Brain Function, Reflex Loops, and Brain Chemistry

Peripheral aVNS modulates various central brain structures involved in autonomic control and in motor control, especially via projections from NTS and nucleus spinalis of the trigeminal nerve, whereas the main therapeutic target is NTS (Figure 1A). In the following, we summarize different methods used to validate this modulation.

(i) Functional magnetic resonance imaging has shown specific modulation of various brain structures following aVNS. These structures include the brainstem and its nuclei NTS and nucleus spinalis of the trigeminal nerve (Chae et al., 2003; Kraus et al., 2007, 2013; Dietrich et al., 2008), and are mostly associated with the higher order relay of afferent (visceral and somatic) VN pathways and networks (Badran et al., 2017). These modulated structures are involved in autonomic regulation, alertness, mood, and well-being control. The brain activation patterns in aVNS were shown to be similar to those during the implanted cervical VNS (Beekwilder and Beems, 2010), indicating favorably similar therapeutic effects of non-invasive and invasive VNS (Mercante et al., 2018b). Most interestingly, activation and deactivation patterns of the brain in response to aVNS over-lasted the effective duration of the stimulation – e.g., by as much as 11 min after cessation of cymba concha stimulation (Frangos et al., 2014) – providing support for the hypothesis of long-lasting sustainable effects of aVNS.
(ii) Evoked far-field brainstem potentials have been reported in response to aVNS since activation of vagal afferents evokes vagus somatosensory potentials in the brain (Fallgatter et al., 2003; Polak et al., 2009). In particular, these potentials reflect the postsynaptic brainstem activity and thus can be regarded as a measure for the brain modulation following aVNS. These brainstem potentials presumably originate in vagal nuclei such as NTS (Fallgatter et al., 2003; Polak et al., 2009) and, again, are similar to evoked potentials from implanted VNS (Nonis et al., 2017). Interestingly, potentials show longer latencies in the elderly than the younger participants (Fallgatter et al., 2004).
(iii) Extracellular recordings in the brain have shown that the percutaneous aVNS decreases spontaneous firing of neurons in the central nervous system – namely, in the central nucleus of the amygdala – by about 50% (Babygirija et al., 2017), which confirms inhibitory effects of aVNS. For instance, the suppression of epileptiform activity was observed via activated firing of NTS neurons in response to aVNS (He et al., 2013). Inhibitory effects of VNS were shown in animal models (Lockard et al., 1990). In particular, motor activity and brainstem neuronal activity were inhibited in cats, as well as the tail-flick reflex in rats. Here a consistent animal alertness was commonly used to find the stimulation threshold indicating inhibitory effects and moreover to ensure comfort and safety of animals.
(iv) Significant changes in the resting electroencephalogram, especially, activation of the mediofrontal theta band, have been observed in response to the implanted VNS (Bonaz and Pellissier, 2016).
(v) Transcranial magnetic stimulation was used to prove increased intracortical inhibition in response to aVNS, most likely due to increased GABAergic activity (Capone et al., 2014).

Auricular vagus nerve stimulation has the potential to improve neuronal plasticity provided that plasticity is maladaptive (Clancy et al., 2012; Lehtimaki et al., 2012). For instance, the invasive VNS paired with auditory stimuli were shown to reverse pathological changes of the cortex in a rat model of tinnitus (Engineer et al., 2011). Since the intact brain plasticity follows different time scales, from quite fast changes within seconds up to slow changes within weeks up to 1 year, aVNS effects can be expected to follow these time scales. In addition, artificial neuromodulation of VN positively affects impaired synchronicity of synaptic activities between and within brain regions as well as overly excited or even deficient reflexes involving central brain pathways (Oleson, 2002; Beekwilder and Beems, 2010). VNS and even aVNS may activate reflexes which inhibit maladaptive reflexes of neuromuscular disorders in humans (Kampusch et al., 2015a) as well as may modulate nociceptive reflexes in rats, namely, facilitate them at low VNS intensities and inhibit at high intensities (Ren et al., 1988, 1993). All these detrimental changes in the brain and reflexes are usually present in chronic diseases such as chronic pain or chronic muscle spasms, which are thus possible application fields of aVNS.

Figure 2B illustrates schematically how the sensory feedback to the brain is lost or impaired – in contrast to intact feedback-loops from Figure 2A – due to the aforementioned detrimental changes in the brain and reflexes, due to maladaptive plasticity and neurodegeneration. Since aVNS recruits sensory fibers to the brainstem (Figure 1), aVNS could be hypothetically expected to mimic the sensory feedback to the brain (Figure 2C), circumventing temporally a lost or impaired feedback. For instance, the invasive and selective stimulation of the cervical VN was used to mimic baroreceptor signals to lower the blood pressure in rats (Plachta et al., 2014, 2016). In analogy, the baroreflex sensitivity was improved by aVNS in humans (Antonino et al., 2017), thus affecting processing of afferent signals in the brain. In addition, aVNS seems to have the potential to restore the missing or altered signaling of intestinal VN afferents (Guiraud et al., 2016).

More importantly, aVNS in Figure 2A could be hypothesized to be followed by sustainable regeneration mechanisms which re-establish the impaired sensory information flow to the brain, as a basis for systemic and sustainable treatment of disease via aVNS. The sustainability of aVNS was demonstrated not only on the brain level, as discussed above with respect to Frangos et al. (2014), but also on physiological level. For instance, the activation of the anti-inflammatory response required only a brief VNS stimulus and lasted for more than 24 h (Olofsson et al., 2015). A sustained antinociceptive effect of aVNS was observed in pain-related gastrointestinal disorders for an extended period of 9 weeks after 3 weeks of treatment (Kovacic et al., 2017), as well as in chronic low back pain for a 3 months follow-up after 6 weeks of treatment (Sator-Katzenschlager et al., 2004).

In response to VNS, endogenous opioid neuropeptides (endorphins) are released in the central nervous system as well as various neurotransmitters (enkephalins and substance P). The release alters properties of neuron membranes, the neuron’s response to synaptic events, and thus alters excitation or inhibition of neuron firing. Consequently, neuronal networks can be potentially reprogramed by VNS, in line with the discussed neuroplasticity. Interestingly, the release of neuropeptides due to aVNS seems to depend on the stimulation frequency (Sator-Katzenschlager and Michalek-Sauberer, 2007). A relatively low frequency of 2 Hz accelerates the release of enkephalin, β-endorphin, and endomorphin. In contrast, a high frequency of 100 Hz selectively increases the release of dynorphin.

Vagus nerve stimulation seems to modulate serotonergic, noradrenergic, and endorphinergic pathways in different brain regions by the relay projections of the vagal afferent nuclei. The associated release of serotonin, norepinephrine, and endogenous opioids is increased while modulating chemically the nociceptive processing in the brain (Lockard et al., 1990; Oleson, 2002; Lehtimaki et al., 2012), the mood and anxiety control, and the pathophysiology of depression (Beekwilder and Beems, 2010; Chakravarthy et al., 2015; Conway and Xiong, 2018; Mercante et al., 2018b). Even aVNS increased norepinephrine levels in rats (Li et al., 2015b). Increased release of noradrenaline in the brain seems to follow VNS, as well as increased amounts of the inhibitory transmitter GABA in the cerebrospinal fluid (Groves and Brown, 2005) and NTS (Mercante et al., 2018b), which potentially leads to VNS-mediated seizure reduction (Clancy et al., 2012) and antidepressant effect (Mercante et al., 2018b). VNS inhibits excitatory glutamate release (Chen et al., 2015c). VNS increases the release of neurotrophic factors as well as stimulates cellular proliferation and neurogenesis in the brain, which have been associated not only with antidepressant effects but also neuronal plasticity, memory, learning and cognitive processes (Mercante et al., 2018b).

In fact, the actual stimulation pathway of aVNS is highly indirect with respect to the distant pathological organ or addressed functions of the body, since aVN has a transmitting but not a processing function. In particular, the stimulation pathway is circuitous. The pathway of the therapeutic electric stimuli starts with action impulses traveling from the peripheral somatosensory aVN endings in the auricle to the brainstem and then to higher order brain structures. Then, the pathway goes either from cranial nerves to the correspondent region of the body or, in analogy, from the brain to the spinal cord and then from spinal nerves to the target body’s region. Both somatic efferent arms (see section “Pain – Experimental Evidence”) and autonomic efferent arms (see section “Modulation of Autonomic Function”) can be expected to be modulated. Distant targets are thus affected via neurological networks or reflexes.

#### Epilepsy

Both VNS and aVNS reduced epileptic seizure activity in rats (He et al., 2009) via activated firing of neurons in NTS (He et al., 2013). Authors in Zagon and Kemeny (2000) suggest a vagally mediated hyperpolarization and thus reduced excitability of cortical neurons that otherwise would be involved in propagation of seizures. VNS decorrelated cortical synchrony and rhythmicity in rats indicating a potentially reduced seizure activity (Nichols et al., 2011). The period of protection from seizure activity persisted beyond the stimulation period (Schachter and Saper, 1998), whereas VNS and aVNS showed similar durations of the anti-seizure effect (Ellrich, 2011).

In humans, aVNS reduced seizure frequency in epilepsy as well as the intensity and duration of seizures, as shown in clinical trials (Rong et al., 2014; Bauer et al., 2016), a case series (Stefan et al., 2012), and reviewed in Beekwilder and Beems (2010) and Shiozawa et al. (2014). The required dosage of antiepileptic drugs was also reduced (Beekwilder and Beems, 2010).

#### Depression

Melatonin secretion in response to aVNS ameliorated the innate depressive behavior in diabetic rats (Li et al., 2014). The antidepressant effect of aVNS was also demonstrated in rats with suggested cardio-inhibitory effects, possibly mediated via the normalization of the hypothalamic-pituitary-adrenal axis hyperactivity in major depression (Liu et al., 2013).

In humans, clinical improvement of depression was shown when treated with the transcutaneous aVNS, whereas depression scores as well as remission rates improved, see clinical trials (Kraus et al., 2007; Hein et al., 2012; Fang et al., 2015; Rong et al., 2016) and a review (Shiozawa et al., 2014).

#### Stroke

Auricular vagus nerve stimulation reduced significantly the infarct volume and improved neurological scores after cerebral ischemia in rats (Ay et al., 2014), whereas only an insignificant reduction in the lesion size was observed after ischemic stroke in rats (Hays et al., 2016). aVNS improved neurobehavioral recovery and upregulated cerebral growth differentiation factor 11 – a rejuvenation factor participating in brain angiogenesis almost in parallel to neurogenesis after stroke – in cerebral ischemia/reperfusion rats (Ma et al., 2016). The transcutaneous aVNS in humans was shown to improve rehabilitation in stroke according to a clinical trial in Capone et al. (2017). These reports indicate aVNS as a potential therapy target for stroke.

Invasive VNS paired with rehabilitative training significantly improved recovery of forelimb function in rats after both intracerebral hemorrhage (Hays et al., 2014) and ischemic stroke within the motor cortex (Hays et al., 2016). Here the sensory inflow along VN potentially provides an associative component to neuronal plasticity in the brain, which enhances plasticity. This may indicate a potential use of aVNS paired with movements to improve cortical representation of movements in the treatment of movement disorders, including the rehabilitation of stroke patients (Porter et al., 2011).

#### Other Disorders

Vagus nerve stimulation improved recovery after traumatic brain injury in rats, based potentially on enhanced neural plasticity and enhanced norepinephrine release (Smith et al., 2005). VNS has shown efficacy in enhancing memory storage processes, as mediated by stimulation of VN afferents in rats (Clark et al., 1998). Furthermore, VNS facilitated visceral pain-related emotional affective memory (Zhang et al., 2012) and enhanced the extinction of conditioned fear when paired with extinction training (Peña et al., 2012) in rats.

In humans, the transcutaneous aVNS is a promising treatment method for autism spectrum disorders and a lot of other psychiatric disorders, as reviewed in Cimpianu et al. (2016) and Jin and Kong (2017), respectively. However, aVNS failed to improve schizophrenia symptoms, as recognized by a clinical trial in Hasan et al. (2015).

In addition, positive effects in Alzheimer’s disease can also be potentially expected because decreased norepinephrine levels are raised by VNS, the levels being essential in maintaining adequate beta amyloid clearance and thus preventing Alzheimer (Beekwilder and Beems, 2010). The neuroprotective role of aVNS was shown in a mouse model of Alzheimer disease (Kaczmarczyk et al., 2017). Interestingly, evoked far-field brainstem potentials after aVNS have been proposed to be used for an early diagnosis of dementias and Alzheimer’s disease, see clinical study in Polak et al. (2007). In particular, latencies of these potentials increase in disease due to degeneration of nervous structures of vagal parasympathetic nuclei.

### Modulation of Nociceptive Processing

The central sensitization represents enhanced excitability of nociceptive pathways due to maladaptive plasticity of the central nervous system [e.g., in the superficial dorsal horn (Todd, 2010)] in response to hyperactivity, inflammation, and neural injury (Latremoliere and Woolf, 2009). This sensitization seems to be a key factor to potentiation in pain sensibility in acute and chronic pain while recruiting previously subthreshold synaptic inputs to excitatory nociceptive pathways. Hyperalgesia can also be produced by illness-inducing agents such as bacterial cell wall endotoxin lipopolysaccharide, whereas these agents activate VN afferents in terms of immunosensation, i.e., sensation of immune-relevant substances (Watkins et al., 1994). The inhibitory and desynchronizing actions of VNS together with enhanced brain plasticity and activated serotonergic pathways (see section “Neurophysiological Evidence – Modulation of Brain Function, Reflex Loops, and Brain Chemistry”), as well as anti-inflammatory effects of VNS (see section “Modulation of Inflammation”), all can be hypothesized to counteract the maladaptive plasticity and inflammation of the central sensitization and thus to counteract pain hypersensitivity. These actions of VNS may potentially contribute to antinociceptive aVNS effects in hyperalgesia, with the aforementioned assumption of similar neurophysiological effects of VNS and aVNS (Beekwilder and Beems, 2010; Mercante et al., 2018b).

Gating mechanisms may become indirectly modulated by aVNS for antinociception, as related to the well-known concordant paresthesia. That is, pain mediated by Aδ and/or C afferents is alleviated by touch or by a simultaneous activation of tactile Aβ afferents from a homotopic site (Ellrich and Lamp, 2005). For instance, hitting a finger can induce pain which can be compensated by rubbing fingers. In particular, the activation of Aβ fibers can inhibit transmission of nociceptive signals in that excited Aβ fibers inhibit presynaptic terminals of Aδ and/or C fibers (Sandkühler, 2000), which is known as activation of inhibitory pain control systems. This leads to wind-down of pain-induced changes in the signal transduction within the spinal cord (Sandkühler, 1996). The electrophysiological data provide evidence that electric stimulation of peripheral Aβ fibers reliably suppresses Aδ fiber nociceptive processing in human volunteers (Ellrich and Lamp, 2005).

Since the stimulated Aβ afferents in the auricle and nociceptive signals along Aδ and/or C afferents do not originate in homotopic sites, we cannot expect a direct activation of gating mechanisms on the spinal level via aVNS. However, since aVN projects via NTS to numerous other brainstem complexes (Nomura and Mizuno, 1984) mutually interacting with spinal regions involved in pain processing (e.g., dorsal horn neurons), an indirect modulation of gating mechanisms can be hypothesized. In addition, aVNS may co-recruit non-vagal auricular nerves (e.g., the great auricular nerve) which end in spinal regions (Mahadi et al., 2019) and thus may directly interfere with gating mechanisms there.

Gating may also refer to blockage of nociceptive signals from ascending peripheral nerves to the brain by descending impulses from the brain (Oleson, 2002). Not only ascending nociceptive stimuli are disrupted but also descending nociceptive signals into the gray matter of the dorsal horn of the spinal column are mitigated (Roberts et al., 2016).

In addition, encephalin-containing interneurons within the spine are proposed to be activated resulting in the inhibition of conduction of pain signals to the brain (Sator-Katzenschlager and Michalek-Sauberer, 2007). Single neuron studies reveal that activation of vagal afferents mostly inhibits nociceptive neurons in the spinal cord in response to noxious stimuli, especially at greater intensities of VNS; e.g., 77% of observed spinothalamic tract neurons were inhibited (Ren et al., 1991). Interestingly, the effective inhibition of nociceptive processing in humans can even outlast conditioning electric stimuli (Ellrich and Lamp, 2005), which may theoretically lead to short-term and even long-term afferent-induced analgesia (Sandkühler, 2000) in response to aVNS (Sator-Katzenschlager et al., 2004).

For effective antinociception using peripheral nerve stimulation, a non-painful stimulus is required to excite rapidly conducting thick myelinated Aβ fibers but not nociceptive slowly conducting thin myelinated Aδ and thin non-myelinated C fibers. Please note that excitation thresholds of Aβ fibers are lower than those of Aδ and C fibers because both myelinization and increasing fiber thickness reduce their threshold (Kaniusas, 2019). Therefore, the nociceptive threshold in different nerves was found to be 5–7 times larger than the detection threshold, whereas near maximal activation of Aβ fibers was suggested to be only 4 times the detection threshold (Ellrich and Lamp, 2005). For comparison, the activity of the least excitable C fiber can be evoked by stimuli with intensities 15–20 times the detection threshold (Ellrich and Lamp, 2005); in line with (Guiraud et al., 2016) stating that the activation threshold of C fibers is 10–100 times greater than that of A fibers.

However, the requirement of the recruitment of non-C-fibers for antinociception in peripheral nerve stimulation contrasts with experimental investigations on the invasive VNS and thus potentially indicates different mechanisms of action in aVNS and in VNS. That is, single neuron studies in rats (Ren et al., 1988, 1991) and targeted pharmacological blunting of C-fibers (Ren et al., 1993) indicate that a low-intensity VNS accelerates discharges of nociceptive neurons in the spine, facilitates nociceptive reflexes and thus produces pronociception, and suggests activation of low-threshold myelinated non-C-fibers of the stimulated afferent VN. In contrast, a high-intensity VNS yields opposite effects of antinociception due to the activation of high-threshold C-fibers but not low-threshold myelinated non-C-fibers of the afferent VN (Ren et al., 1993).

In general, stimulated VN afferents can exert both inhibitory and excitatory modulation of the nociceptive processing in spinal and supraspinal regions, in central and peripheral pain pathways (Berthoud and Neuhuber, 2000; Busch et al., 2012; Napadow et al., 2012). Low stimulus intensities applied in the afferent invasive stimulation of VN tend to induce pronociception (Ren et al., 1988; Ness et al., 2000) while high intensities – still non-noxious but perceivable stimuli – tend to induce antinociception (Chakravarthy et al., 2015). In line with (Randich and Gebhart, 1992), antinociceptive inhibiting effects of VNS begin to counteract pronociceptive facilitatory influences of VNS with increasing VNS intensity. However, some controversy exists concerning pronociception in response to the afferent stimulation of VN (Busch et al., 2012). Some human subjects with implanted VNS have even shown U-shaped thermal pain thresholds with increasing VNS intensity (Ness et al., 2000). Authors in Laqua et al. (2014) report both antinociception and pronociception in response to aVNS in about 70 and 30% of healthy subjects, respectively, accounting this to individual sensitivity. As a hypothesis supported by the authors, low and high intensity VNS may potentially activate different circuits in the brainstem.

Furthermore, stimulation of vagal afferents with the subsequent neuromodulation of NTS (Figure 1A) is hypothesized to underlie the antinociceptive effects of VNS (Napadow et al., 2012). This is because NTS acts as an integrating station for nociceptive afferent stimuli (Boscan et al., 2002) and as a relay station and inputs to higher brain regions, which process and modulate different aspects of pain (Saper, 2002). Furthermore, spinal regions processing pain project to NTS (Todd, 2010), such as the dorsal horn neurons (Mahadi et al., 2019). For instance, an electrical stimulation of NTS was shown to inhibit nociceptive responses at the spinal cord level (Du and Zhou, 1990), whereas a local anesthetic block of NTS eliminated the inhibitory effect of VN stimulation on the nociceptive tail flick reflex in rats (Randich and Aicher, 1988). Authors in Napadow et al. (2012) have shown less antinociceptive effects in an auricular non-VN stimulation as compared to the auricular VN stimulation mediated by NTS.

#### Pain – Experimental Evidence

In animals, VNS attenuated heat-induced and formalin-induced pain in rats (Bohotin et al., 2003). aVNS increased paw withdrawal threshold in rats and attenuated baseline firing of neurons in the central nucleus of the amygdala and spinal cord neurons by about 50%, which may account for the modulation of pain responses (Babygirija et al., 2017). VNS was shown to inhibit cortical spreading depression, a propagating wave of depolarization that underlies migraine aura and thus triggers headache in rats (Chen et al., 2015c). Interestingly, the non-invasive VNS on the neck demonstrated the potential to alleviate trigeminal allodynia in rats (Oshinsky et al., 2014). This type of VNS decreased levels of the extracellular glutamate in the trigeminal nucleus caudalis, a neurotransmitter that increases with trigeminal pain.

In humans, the electrical stimulation of auricular regions including aVN increased electrical pain thresholds by 30–50% in about half of the studied healthy subjects (Johnson et al., 1991), increased mechanical and pressure pain thresholds (Beekwilder and Beems, 2010; Busch et al., 2012), as well as increased pressure pain thresholds and decreased pain ratings under sustained application of painful heat (Ellrich et al., 2011). Once again, predominant inhibitory effects of aVNS are highlighted by these cited studies.

#### Pain – Clinical Evidence

Antinociceptive effects of aVNS were shown in numerous clinical trials: chronic cervical pain (Sator-Katzenschlager et al., 2003), chronic low-back pain (Sator-Katzenschlager et al., 2004), acute pain during *in vitro* fertilization (Sator-Katzenschlager et al., 2006), postoperative pain after laparoscopic nephrectomy (Likar et al., 2007), postoperative pain after tonsillectomy (Kager et al., 2009) – as also supported by a review (Cho et al., 2015) – postoperative pain after hysterectomy (Tsang et al., 2011), headache syndrome (Busch et al., 2012), high-frequency and chronic migraine (Straube et al., 2015), acute migraine (Garcia et al., 2017), chronic abdominal pain-related functional gastrointestinal disorders (Kovacic et al., 2017), and chronic pelvic pain (Napadow et al., 2012). A few case studies support antinociceptive effects of aVNS, namely, in postoperative pain (Szeles et al., 2001) – as also supported by a review (Liu et al., 2015) – chronic muscle pain in dystonia (Kampusch et al., 2015a), pain in peripheral arterial occlusive disease (Payrits et al., 2011), pain in primary Raynaud’s syndrome (Schlager et al., 2011), labor pain (Grünberger et al., 2005), and finally in diverse musculoskeletal pain disorders (unpublished data by our group in Vienna).

The percutaneous aVNS significantly reduced opioid intake, such as tramadol (Sator-Katzenschlager et al., 2003, 2004), remifentanil (Sator-Katzenschlager et al., 2006), morphine-hydrochloride (Likar et al., 2007), naproxen and tramadol (Kager et al., 2009), all latter studies are clinical trials, and morphine, as reviewed in Liu et al. (2015). aVNS reduced anesthetic requirements in response to noxious electrical stimulation, as shown in a clinical trial (Greif et al., 2002) and reduced analgesic medication intake after abdominal and accident/trauma surgery, as shown by a case series in Szeles et al. (2001).

However, the percutaneous aVNS in the following clinical trials failed to show reduced acute pain and reduced analgesic consumption in the perioperative setting of the third molar tooth extraction (Michalek-Sauberer et al., 2007), as well as aVNS failed to reduce postoperative pain and opioid consumption in women undergoing elective gynecological laparoscopy (Holzer et al., 2011).

In general, results for aVNS are controversial in acute pain but are rather consistent in chronic pain (Sator-Katzenschlager and Michalek-Sauberer, 2007). This suggests that aVNS may be rather a long-term adjunctive therapy for treating chronic pain than an acute treatment (Clancy et al., 2012). In fact, chronic pain and acute pain are two different processes. Acute pain is a physiological process with well-defined anatomical pathways underlying the perception of nociception. In contrast, chronic pain is a pathological state associated to the complex rewiring of circuitries of the central nervous system, reported as maladaptive plasticity (Colangelo et al., 2014).

Auricular vagus nerve stimulation decreased symptoms of acute opioid withdrawal – related to heroin, methadone and others – after just 1 h of stimulation and allowed for an effective transition to non-opioid assisted medication therapy in some patients, as illustrated by a retrospective case series in Miranda and Taca (2017). aVNS was shown to be associated with temporary relief of withdrawal symptoms in heroin-addicted subjects on and after treatment in the first 3 days, see a case series in Wen et al. (1978).

### Modulation of Inflammation

Inflammation processes are governed through interrelated humoral and neural reflex pathways (Tracey, 2009; Miller and Raison, 2015). In particular, chronic inflammation is based on deregulation of metabolic and immune functions, whereas the imbalance between pro-inflammatory and anti-inflammatory cytokines seems to be decisive in disease progression (Neurath, 2014). Abnormal and chronic inflammation is implicated in, causes and advances, numerous wide-spread chronic diseases as diabetes mellitus and is, for example, a major hindering factor in effective neuroprotection in the brain, e.g., after stroke.

The vagus nerve provides a first-line defense against infection and inflammation in the periphery to restore homeostasis via conducting information to/from the brain to regulate the immune system. VN is a major component of the neuroendocrine-immune axis (Bonaz and Pellissier, 2016). For instance, even fever, as a brain-mediated response, is signaled to the brain via afferent VN responding to peripheral proinflammatory cytokines, in addition to blood-borne routes for the fever’s signaling (Hansen et al., 2001).

The parasympathetic outflow along VN, i.e., activation of the parasympathetic system, has only anti-inflammatory effects. This was shown by the inverse relationship between VN-mediated parasympathetic markers of the heart rate variability (HRV) and inflammatory markers (Thayer and Fischer, 2008). In contrast, the sympathetic nervous system may have both pro-inflammatory and anti-inflammatory effects.

In general, VN is involved in mainly three reflex pathways with a clear anti-inflammatory role:

(i) The anti-inflammatory hypothalamic-pituitary-adrenal axis. Here afferent VN fibers sense the level and location of injury/infection in that pro-inflammatory cytokines and/or endotoxins activate VN endings. Somatotopic maps in NTS become activated. Consequently, special neurons in hypothalamus activate the release of hormone adreno-corticotrophin by the hypophysis, stimulating the release of glucocorticoids by the adrenal glands to decrease peripheral inflammation.
(ii) The anti-inflammatory vago-vagal reflex, known also as the cholinergic anti-inflammatory pathway (Borovikova et al., 2000; Tracey, 2007). Here infection-activated afferent VN fibers synapse with and generate an outflow along efferent VN fibers releasing acetylcholine at their synaptic endings. The acetylcholine binds to surface receptors of macrophages and suppresses the production and release of pro-inflammatory cytokines by these macrophages.
  Interestingly, the tonic neural activity of this cholinergic anti-inflammatory pathway is essential because, when it is impaired, over-inflammation results with an unrestrained cytokine release damaging tissue (Mercante et al., 2018a). From this perspective, VNS enhances the activity of immune-related neural circuits and confers protection of the human body.
(iii) The splenic sympathetic anti-inflammatory pathway. Here the infection-activated afferent VN yields outflow along the efferent VN which stimulates the adrenergic sympathetic nerve in the spleen, releasing norepinephrine at its endings. Then norepinephrine binds to splenic lymphocytes and leads to acetylcholine release by lymphocytes, whereas acetylcholine, in turn, inhibits the release of pro-inflammatory cytokines by splenic macrophages.

Thus, the innate immune system is subjected to a closed-loop reflex modulation via afferent VN fibers, as illustrated in Figure 2A, whereas the activity of the efferent VN maintains homeostasis by limiting pro-inflammatory responses and avoiding immunosuppression. For instance, the role of VN is proposed in informing the brain about peripheral inflammation related to coronary artery disease and in actively modulating the disease related inflammation (Gidron et al., 2006).

An artificial VNS has been shown to harness this natural reflex (Figure 2C). The modulation of VN results in decreased pro-inflammatory and increased anti-inflammatory cytokines, which is effective in suppression of over-inflammation, prevention of tissue injury, and improved survival. For instance, aVNS reduced pro-inflammatory cytokines, as shown in a clinical trial (Stavrakis et al., 2015), and increased norepinephrine levels, as reviewed in Beekwilder and Beems (2010), which supports anti-inflammatory aVNS effects.

In particular, VNS was shown to rebalance the working point of autonomic regulation of the immune system into a protective range avoiding pro-inflammatory responses and, on the other hand, avoiding immunosuppression (Tracey, 2009). Here the working point is defined as the magnitude of innate immune responses relative to the infection or injury stimulus. Chronic changes can unfavorably increase or decrease the working point with the resulting overshooting immune response (with tissue damage, sepsis, or even death) or immunosuppression (with secondary infections), respectively.

In animals, VNS had favorable effects on rheumatoid arthritis in rats (Koopman et al., 2017). VNS reduced surgery-induced intestinal inflammation and improved postoperative intestinal transit in mice, supporting the anti-inflammatory effect of VNS (Matteoli et al., 2013). In addition, VNS prevented the development of shock in rats through inhibited synthesis of the tumor necrosis factor (cytokines) (Borovikova et al., 2000). aVNS was shown to be efficient in mice with lethal endotoxemia or polymicrobial sepsis while reducing systemic tumor necrosis factor due to anti-inflammatory aVNS effects (Huston et al., 2007). aVNS suppressed lipopolysaccharide-induced inflammatory responses in endotoxemic rats through reduced pro-inflammatory cytokines, indicating that aVNS modulates the immune function through the cholinergic anti-inflammatory pathway (Zhao et al., 2012).

In humans, potential therapeutic applications of aVNS are related to chronic inflammatory conditions. These are rheumatoid arthritis, see a clinical trial in Becker (2007), inflammatory bowel disease (Crohn’s disease, ulcerative colitis), and postoperative ileus in order to restore intestinal homeostasis, as reviewed in Tracey (2007), Marshall et al. (2015), and Bonaz and Pellissier (2016).

### Modulation of Autonomic Function

Vagus nerve stimulation and aVNS are followed by a broad physiological multi-level response, as already outlined above. The stimulation leads to systemic autonomic effects in terms of the parasympathetic stimulation of the body (He et al., 2016). The antagonistic action of the activated parasympathetic system over the sympathetic is one of the expected therapeutic mechanisms of aVNS, as, for instance, reflected by estimation of the shift of the ANS activity toward parasympathetic dominance with reduced sympathetic contribution (Clancy et al., 2014; Kampusch et al., 2015a).

That is, the sympathovagal balance – or the activity of ANS (HRV, 1996; Billman et al., 2015) – seems to improve in response to aVNS, as estimated by its standard measure, HRV (Gbaoui et al., 2008; Kaniusas et al., 2008; Kampusch et al., 2015b; Gomolka et al., 2018). For instance, the parasympathetic tone can be estimated from HRV during respiration cycle (HRV, 1996). While a shift toward parasympathetic predominance was indicated in aVNS by a decreased ratio of low-frequency to high-frequency (LF/HF) components of HRV (Deuchars et al., 2017), muscle sympathetic nerve activity (derived by microneurography), as a marker for the total sympathetic outflow, decreased (Clancy et al., 2014). However, authors in De Couck et al. (2016) and Antonino et al. (2017) report on partly diverging results on HRV in response to aVNS.

Baseline values seem to have an influence on the autonomic response due to aVNS or VNS. For instance, a higher resting ratio LF/HF predicted its greater decrease during aVNS in healthy humans, implying that humans with higher sympathetic activity are subjected to a stronger aVNS effect (Clancy et al., 2014). However, the invasive VNS in epilepsy patients showed an inverse behavior, in that a higher parasympathetic activity, e.g., a higher HF level, led to a better therapeutic outcome of VNS (Liu et al., 2017).

### Modulation of Metabolic Syndrome

Reduced activity of VN, especially, decreased parasympathetic and increased sympathetic activity, are hypothesized to underlie metabolic syndrome (De Couck et al., 2011). Therefore VNS and aVNS can be expected to reduce risks of metabolic syndrome that includes obesity, elevated glucose levels, diabetes, elevated blood pressure, and increased inflammation (Pavlov and Tracey, 2012).

In animals, VNS is associated with the weight loss, reduced fat mass, and decreased appetite (Sobocki et al., 2005; Browning, 2010; Banni et al., 2012). For instance, VNS decreased weight gain, food consumption, and sweet craving in adult obese minipigs in the context of morbid obesity (Val-Laillet et al., 2010; Cork, 2018). aVNS reduced body weight in rats and the amount of the white adipose tissue in viscera (Li et al., 2015b), indicating an important role of VN in obesity management. Interestingly, an imbalance in ANS with predominant parasympathetic activity is suggested to cause obesity in rats (Barella et al., 2014). aVNS showed antidiabetic effects in rats through triggered secretion of melatonin involved in the regulation of glucose metabolism (Wang S. et al., 2015) and through reduced diabetic cellular injury (Mei et al., 2013). This is because VN is strongly involved in the glucose metabolism and insulin secretion (Barella et al., 2014).

In humans, a reduction of body weight and body mass index was observed in chronically obese females in response to aVNS in a clinical trial (Schukro et al., 2013). This is in line with a case series in Szeles et al. (2001) in which the tendency of aVNS to reduce body weight and body mass index in adipose patients was observed. aVNS seems to affect appetite and satiety, inhibiting food intake and thus body weight (Schukro et al., 2013), as well as seems to affect glucose metabolism, as reviewed in Guiraud et al. (2016), with a potential impact on the reward system in the brain. Here the arcuate nucleus of the hypothalamus seems to play an important role in mediating the satiety in response to aVNS. In addition, aVNS and the associated raised serotonin levels have been shown to increase the tone in smooth muscles of the stomach and to enhance intestinal motility, suppressing appetite and contributing to the loss of body weight in obese people, as shown in a clinical trial in Richards and Marley (1998) and reviewed in Ergene and Tan (2006).

In obese humans, the food signaling from the stomach is altered as a consequence of the reduced sensitivity of small intestinal VN afferents, so that aVNS seems to have the potential to restore this missing or altered signaling, as reviewed in Guiraud et al. (2016). This is in close analogy with the presented hypothesis in Figure 2.

Human studies indicate that low VN activity may underlie elevated glucose levels, whereas the transcutaneous aVNS significantly reduced the 2-h glucose tolerance (Huang et al., 2014). aVNS was shown to decrease glycated hemoglobin, blood urea nitrogen, serum creatinine, total cholesterol, and aspartate transaminase in patients with type 2 diabetes mellitus (Ju et al., 2014). Both referenced clinical trials show the potential of aVNS as a preventive treatment for pre-diabetes and as a complementary treatment of diabetes patients, respectively.

### Modulation of Cardiovascular Effects

Vagus nerve stimulation effects – especially effects of the parasympathetic modulation (Olshansky et al., 2008) – on hemodynamic and cardiovascular control have been demonstrated (Ness et al., 2000; De Ferrari and Schwartz, 2011). However, consistent and significant changes in hemodynamic measures are usually absent at relatively low VNS intensities (e.g., below levels used for seizure control in humans) that are accompanied by pronociception, whereas hemodynamic measures are invariably altered at higher VNS intensities, especially at intensities with antinociceptive effects (Ness et al., 2000).

In animals, invasive VNS attenuated mean arterial blood pressure and reduced the number of arrhythmia episodes in hypertensive rats (Annoni et al., 2015), whereas it increased coronary flow in dogs (Tiedt and Religa, 1979). aVNS elicited cardiovascular responses in rats, characterized by a lowered blood pressure (Mahadi et al., 2019) and heart rate (Gao et al., 2007). Cardiovascular inhibition in rats due to aVNS was also shown in Gao et al. (2010), where reduced blood pressure and heart rate were paralleled by excitation of cardiac-related neurons in NTS. Similarly, vasodepressor and bradycardic effects of VNS were attenuated by local anesthetic blockage of NTS (Randich and Aicher, 1988). In dogs, the protective anti-arrhythmic role of VNS was shown in terms of preventing ventricular fibrillation after healed myocardial infarction (Vanoli et al., 1991). Interestingly, there are indications that a strong invasive VNS tends to facilitate atrial fibrillation, whereas a moderate VNS tends to inhibit atrial fibrillation without arrhythmogenic risks (Chen et al., 2015a).

In humans, an increased cerebral blood flow was found in certain brain regions associated with afferent pathways of VN on acute activation of VNS, as shown in a clinical trial in Conway et al. (2006). aVNS was also reported to increase the velocity of cerebral blood flow in the supratrochlear artery and the middle cerebral artery in single patients (Széles and Litscher, 2004). aVNS decreased the carotid-femoral pulse wave velocity, as shown by a clinical trial in Hackl et al. (2017).

Auricular vagus nerve stimulation reduced the systolic blood pressure over time in patients with impaired glucose tolerance (Huang et al., 2014) and in patients with coronary artery disease (Zamotrinsky et al., 2001), both studies are clinical trials. The trial in Zamotrinsky et al. (2001) showed markedly reduced sympathetic inflow to the heart in patients with coronary artery disease treated with the percutaneous aVNS. In general, aVNS decreased the heart rate, the systolic blood pressure, and improved the left ventricular diastolic filling and ejection fraction. A clinical trial in Badran et al. (2018b) demonstrated different changes in the heart rate in response to various aVNS parameters.

As shown in the following clinical trials in humans, aVNS suppressed atrial fibrillation in patients with paroxysmal atrial fibrillation (Stavrakis et al., 2015) and improved cardiac function in patients with coronary artery disease via upregulation of the protective heat shock proteins and reduction of the heart rate (Afanasiev et al., 2016). Furthermore, aVNS led to relief of anginal symptoms in coronary artery disease patients who underwent coronary artery bypass grafting operations, see a clinical trial in Zamotrinsky et al. (1997). Here aVNS resulted in diminution of biochemical myocardial signs of the disease, an increase in the heart’s tolerance of operative reperfusion damage, and a reduced need for vasodilators.

Heart failure is another relevant condition for neuromodu-lation (Stavrakis and Po, 2015), characterized by a chronic autonomic imbalance with reduced VN activity and accelerated sympathetic activity (Olshansky et al., 2008; De Ferrari and Schwartz, 2011; Byku and Mann, 2016). In animals, VNS was proposed as a potential treatment of heart failure and angina pectoris (Bilgutay et al., 1968). VNS improved the long-term survival of rats with chronic heart failure through the prevention of pumping failure and cardiac remodeling (Li et al., 2003). VNS improved HRV and baroreflex sensitivity in a dog model of heart failure, in close association with a reduction in the expression of inflammatory cytokines and attenuated development of heart failure (Zhang et al., 2009). Beneficial effects of VNS may include anti-adrenergic effects at central and peripheral levels, anti-apoptotic effects, an increase in nitric oxide (NO), and anti-inflammatory effects (Olshansky et al., 2008; De Ferrari and Schwartz, 2011). In humans, aVNS acutely improved the spontaneous cardiac baroreflex sensitivity, as shown by a clinical trial (Antonino et al., 2017). A wealth of clinical studies shows that increased vagal activity reduces the risk of ischemia-related mortality, see clinical trial in De Ferrari et al. (2010). Thus, heart failure may favorably be treated by the invasive VNS in humans (De Ferrari et al., 2010) through blunted sympathetic activity and suppressed proinflammatory cytokines, as reviewed in Guiraud et al. (2016). However, more recent clinical trials in heart failure failed to show efficacy of VNS with respect to reduced rates of death (Gold et al., 2016). Since stimulation effects of the invasive VNS and aVNS were shown to be similar in reviews of Beekwilder and Beems (2010) and Mercante et al. (2018b), it can be hypothesized that aVNS could be favorably applied in heart failure, as reviewed in Wang Z. et al. (2015) and He et al. (2016).

Microcirculation is also potentially subjected to aVNS. Release of vasodilating NO may be involved in mediating cardiovascular responses to aVNS, as mentioned in Sator-Katzenschlager and Michalek-Sauberer (2007), leading to enhanced peripheral perfusion. NO acts also as a ubiquitous sympatholytic and vagotonic messenger for intracellular signaling in central and peripheral regions of the autonomous control, e.g., the neuronally mediated NO increases NTS neuronal activity and inhibits central sympathetic outflow (Chowdhary and Townend, 1999). It may be hypothesized that the neuronally mediated NO can be released through the gracile nucleus-thalamic pathway in response to the afferent input and/or input from the dorsal horn neurons (Rong and Ma, 2011), which were reported to be activated by projections from the great auricular nerve co-stimulated by aVNS (Mahadi et al., 2019). In addition, since the anti-inflammatory effects of VNS on the vascular level prevented an inflammation-related inhibition of NO release in vascular endothelium (Chapleau et al., 2016), aVNS with its anti-inflammatory effects may be hypothesized to improve vascular function through endothelial NO release.

Following the modulation of ANS by aVNS, the capillary-venous oxygenation in deep tissues under the skin (at about 8 mm) was observed to increase in diabetic patients, indicating aVNS effects on vasotonus, as indicated by our clinical trial (Kaniusas et al., 2015). An absolute increase in the mean skin temperature was observed in response to aVNS in single patients with peripheral arterial disease and chronic diabetic wounds, whereas a widening and shrinking of high and low temperature skin regions, respectively, was observed, as shown by a case series in Szeles et al. (2013) and our clinical trial (Clodi, 2016).

Therefore, aVNS may potentially improve healing of diabetic wounds. Here the potential contributors are an increased local blood perfusion and reduced local inflammation, as indicated by our clinical trial in Kaniusas et al. (2015) and Thomas (2017), an increased temperature of the wound region, see our clinical trial in Clodi (2016) and a case series in Szeles et al. (2013), as well as a normalized sympathovagal balance toward parasympathetic predominance due to aVNS, as reviewed in Clayton and Elasy (2009) and shown by our clinical trial (Kampusch et al., 2015b). aVNS in combination with platelet rich fibrin was observed to contribute to avoidance of amputations due to chronic lower leg ulcers resistant to conventional treatment in clinical routine, see a case series in Leitner et al. (2015). Thus, it may be hypothesized that a multimodal therapeutic concept including aVNS contributes to a better prognosis of patients with diabetic foot syndrome and facilitates a conversion of chronic into active wounds (with their possible closure) avoiding debilitating amputations. Clearly, further studies are certainly needed to reveal underlying mechanisms and prove clinical significance of aVNS in the wound treatment.

Auricular vagus nerve stimulation significantly improved symptoms in peripheral arterial occlusive disease through significantly increased pain-free walking distance, as shown in a case series in Payrits et al. (2011). However, a clinical trial in Hackl et al. (2017) has shown a significant increase in the initial walking distance in both verum and control groups, whereas the total scope of the Walking Impairment Questionnaire significantly improved only in the verum group.

In patients with the primary Raynaud syndrome, no significant changes in the skin perfusion and temperature were observed in response to aVNS, as shown by a case series in Schlager et al. (2011); however, aVNS still reduced attack frequency in these patients.

### Modulation of Cardioprotective Effects

Ischemic myocardial infarction is resolved by a timely reperfusion of the occluded coronary artery, which results in a robust reduction of the acute mortality but also results in an increased incidence of chronic heart failure. Paradoxically, reperfusion itself causes injury to the tissue, known as myocardial reperfusion injury. In particular, the reperfusion of the jeopardized myocardium is connected with contractile dysfunction, adverse left ventricular remodeling, cell necrosis, and inflammatory response (potentially leading to remote vascular injury). The severity of the myocardial reperfusion injury is tightly related to parasympathetic hypoactivity and sympathetic hyperactivity (Florea and Cohn, 2014), which is qualitatively in line with the discussed autonomic imbalance in heart failure in humans. Therefore, reestablishment of the sympathovagal balance via the parasympathetic activation – using VNS or aVNS – seems to be a potential therapeutic strategy (Byku and Mann, 2016).

In a rat model, the invasive VNS reduced the infarct size and ameliorated myocardial dysfunctional vasoconstriction and vasodilatation after myocardial ischemia and reperfusion (Zhao et al., 2013). These cardio-protective effects of VNS were associated with a marked reduction in inflammatory markers, i.e., pro-inflammatory cytokines (Calvillo et al., 2011). VNS applied during acute myocardial ischemia markedly reduced arginase expression in the myocardium and aorta (as induced by ischemia and reperfusion), and further elucidated the cardio and vascular protective effect of VNS (Kiss et al., 2017). In a swine model, VNS reduced infarct size, ventricular fibrillation incidence, and improved cardiac function in the course of strengthened parasympathetic activity (Shinlapawittayatorn et al., 2014).

Cervical VNS was particularly shown to release NO by neurons (and potentially by myocardial cells) in the heart of rabbits, in addition to the basal NO produced by endothelial cells (Brack et al., 2009). NO is suggested to enhance the cardiac vagal control while buffering sympathetic activity in terms of VNS-mediated cardioprotection (Chowdhary and Townend, 1999; Brack et al., 2007). The released NO may account for a number of VNS effects in the heart, enhancing bradycardia (Chowdhary and Townend, 1999; Conlon and Kidd, 1999) and ventricular force control (Brack et al., 2009). There is evidence that NO mediates the anti-fibrillatory effect of VNS on ventricles (Brack et al., 2007) and atria (Stavrakis et al., 2013).

Interestingly, an intermittent but not a continuous VNS attenuated the sympathetic tone and thus reduced the infarct size after myocardial ischemia and reperfusion; in fact, there is a strong association between sympathetic stress and infarct size (Buchholz et al., 2014). In particular, the continuous VNS in rabbits produced bradycardia and thus long times of the diastolic ventricular filling, leading to increased atrial and ventricular volumes, with the consequence that pressures within atria and ventricles increase. Loading conditions increase, ventricular and atrial walls are stretched, which, in turn, activate embedded vagal afferents. This leads to sympathetic compensatory neural reflexes, potentially responsible for the increase of the infarct size (Buchholz et al., 2014). In contrast, the intermittent VNS was not able – or was not intense enough – to increase these loading conditions but antagonized intermittently the sympathetic system, reducing the infarct size; compare with the aforementioned effects of strong VNS versus moderate VNS to cease atrial fibrillation.

In analogy with VNS, aVNS was shown to reverse cardiac remodeling, improve cardiac function, and reduce infarct size in dogs with myocardial infarction (Wang et al., 2014a, b). In particular, left atrial and left ventricular dilatation were reduced, left ventricular end-systolic and end-diastolic dimensions were reduced, left ventricular contractile and diastolic functions improved, ejection fraction improved, and interstitial fibrosis and collagen degradation attenuated. Authors in Yu et al. (2012) showed that aVNS was able to reverse atrial remodeling in dogs, increase the effective refractory period in atria, and thus to inhibit inducibility of atrial fibrillation.

### Modulation of Psychometric Functions

Auricular vagus nerve stimulation was shown to improve different psychometric functions – as illustrated by the following clinical trials – including well-being, alertness, cognitive performance while decreasing negative mood (Kothe, 2009), in line with improvement of well-being in Kraus et al. (2007). Subjective well-being in terms of reduced nausea and tiredness was improved by aVNS during and after oocyte aspiration (Sator-Katzenschlager et al., 2006). Well-being, activity, and sleep were improved in patients with chronic cervical pain (Sator-Katzenschlager et al., 2003) and with low back pain (Sator-Katzenschlager et al., 2004). Anxiety was reduced in chronic pelvic pain patients (Napadow et al., 2012). A case series in Szeles et al. (2001) reported aVNS to improve the general personal and constitutional condition after abdominal and accident surgery. Preliminary data suggest that aVNS positively affects sleep, as shown by a clinical trial in Becker (2007) and a case series in Szeles et al. (2010). aVNS may also attenuate postoperative cognitive dysfunction in elderly patients, as hypothesized in Xiong et al. (2009).

The results of the following clinical trials show that the transcutaneous aVNS seems to improve associative memory performance in older individuals, even after a single treatment session (Jacobs et al., 2015). aVNS enhanced divergent thinking due to a speculated increase in GABA levels in the brain (Colzato et al., 2018) and enhanced response selection during action cascading processes indicating the important role of the increased GABA and norepinephrine concentrations (Steenbergen et al., 2015). Relaxing and sedating effects of aVNS were shown in Litscher et al. (2007) based on a reduced EEG-bispectral index. These positive psychometric effects of aVNS may be potentially ascribed to improved control by ANS and to accelerated parasympathetic activity.

### Modulation of Other Functions

Vagus nerve stimulation effects on respiration and gastrointestinal control have been demonstrated (Ness et al., 2000). For instance, aVNS was shown to strengthen respiratory sinus arrhythmia – i.e., the respiratory-induced and vagus-mediated change in the cardiac interval divided by the tidal volume (La Marca et al., 2009) – which is proportionally related to the parasympathetic activity.

The rationale for gastrointestinal control by VNS or aVNS is that the vagovagal neurocircuitry modulates the enteric nervous system and thus influences gastric functions (Bonaz and Pellissier, 2016). For instance, VNS in rats accelerated gastric emptying, caused a greater relaxation or dilation of the pyloric sphincter, and increased antral contraction amplitude, peristaltic velocity but not its contraction frequency (Lu et al., 2018). Gastric contraction in rats resulted also in response to aVNS (Gao et al., 2007). aVNS ameliorated burn-induced gastric dysmotility and improved its emptying in rats (Li et al., 2015a). Interestingly, cardiovascular and gastric responses were abolished by blockade of vagal transmission using the muscarinic receptor blocker atropine, which highlights the involvement of VN in both responses and in aVNS (Gao et al., 2007).

Auricular vagus nerve stimulation was highly efficient in treating a single case of refractory dystonia, whereas preliminary data suggest both an immediate effect of aVNS on motor control loops and even sustainable long-term effects on ANS, see a case study in Kampusch et al. (2015a). aVNS improved spinal mobility in pain affected patients, see a case series in Kletzl (2012). aVNS reduced tinnitus severity when paired with tailored sound therapy (Lehtimaki et al., 2012) and reduced sympathetic dominance in tinnitus patients (Ylikoski et al., 2017), both are case series. Here neuroplastic effects of aVNS resolving pathological plasticity in the cortex are potentially involved, as reviewed in Clancy et al. (2012). aVNS was shown to reduce delivery time besides reduced labor pain, as shown in a case series in Grünberger et al. (2005). aVNS could also be beneficial to treat chronic hiccups in response to an altered VN activity, whereas VN is involved in the afferent and efferent limbs of the hiccup reflex, as reviewed in Clancy et al. (2012). Likewise, balancing of VN activity via aVNS could also be beneficial in some conditions like chronic cough in order to reduce over-activation of vagal respiratory afferents (Clancy et al., 2012).

## Limitations

A few indirect but rather seldom unwanted effects can also be triggered by aVNS due to afferent-efferent vagal reflexes, with NTS as a potential intermediate stage. The Arnolds ear-cough reflex is the most dominant reflex, in which mechanical irritation of the auricular skin with embedded aVN may cause cough. Other reflexes are ear-gag reflex, ear-lacrimation reflex, ear-syncope reflex, and vaso-vagal reflex. These vegetative reflexes can occur with the respective incidence up to a few percent in the general population (Tekdemir et al., 1998; Ellrich, 2011; Napadow et al., 2012).

In the transcutaneous aVNS, relatively large surface electrodes yield diffuse stimulation fields. Therefore, not only aVN but also other non-vagal fibers in the ear can be expected to be stimulated (Figure 1A). Relatively strong currents and good electrode contacts are required for the current stimuli to circumvent the skin barrier of the ear and still stay suprathreshold in regions innervated by aVN; however, the transcutaneous aVNS is considered as safe (Badran et al., 2018b). The remaining side effects are mostly minor – as related to invasive VNS (Liporace et al., 2001) – and may include headache, pain and skin irritation at the stimulation site, and dizziness (Mertens et al., 2018). In contrast to the transcutaneous aVNS, the auricular needle electrodes in the percutaneous aVNS and the resulting focused stimulation favor precise and local stimulation of aVN endings. Here the electrode contact impedance is lower and more reproducible, favoring a low current stimulation. Minor side effects are local skin irritation, local bleeding, local pain, and dizziness. The clinical incidence of skin irritation and inadvertent bleeding can be reduced down to only 0.05% using a transillumination technique of the ear (Kaniusas et al., 2011), which visualizes auricular vessels to avoid random placement of needles (Roberts et al., 2016).

A special recognition is required on the recent controversy on whether the stimulation effects of aVNS are due to the recruitment of aVN and on the true anatomical location of aVN. The literature on the definite innervation of the auricle is very sparse and is usually based on often cited findings in Peuker and Filler (2002), which unfortunately show some inconsistencies with respect to aVN innervation regions (Burger and Verkuil, 2018). These uncertainties impede a proper interpretation of stimulation effects while an optimal target for aVNS is still under debate (Badran et al., 2018a; Burger and Verkuil, 2018).

Auricular vagus nerve stimulation is typically performed at the tragus or (cavum, cymba) concha (Yakunina et al., 2016). However, some approaches cover larger areas of the auricle (Sator-Katzenschlager and Michalek-Sauberer, 2007) with the potential to stimulate concomitantly a few more auricular nerves in addition to aVN, especially the great auricular nerve (with connections to the spinal cord) or the auriculotemporal nerve (connecting to the nucleus spinalis of the trigeminal nerve). Due to a possible co-activation of the great auricular nerve, it may make sense to study in future the activation of spinal cord sites, e.g., dorsal horn activity (Deuchars et al., 2017). However, current imaging studies in humans are usually focused on VN-activated brain stem nuclei and their projections (Yakunina et al., 2016). In rats, as reported recently in Mahadi et al. (2019), tracing of the transcutaneous stimulation at the tragus labeled the dorsal horn of the cervical spinal cord. Here, a central sympathoinhibition by up to 36% was observed in response to the stimulation, as mediated at least in part through sensory afferent projections to the spinal cord and despite of only sparse labeling of NTS, the termination site of aVN (Figure 1A). Authors in Mahadi et al. (2019) also suggest that the tragus stimulation can indirectly influence brainstem regions involved in the sympathetic control via the spinal cord and even suggest an indirect innervation of NTS by recruited aVN via the spinal cord.

It is questioned if the tragus includes aVN endings or only non-vagal endings, such as the great auricular nerve and the auriculotemporal nerve (Badran et al., 2018a; Burger and Verkuil, 2018). A potential recruitment of these nerves would suggest that mechanisms may be involved for tragal stimulation beyond those anticipated for the sole aVN stimulation. Only the cymba concha was found so far to be solely innervated by aVN (Peuker and Filler, 2002) with the associated maximum activation of vagal projections in NTS during stimulation, as compared to tragus, cavum concha, or earlobe stimulation (Yakunina et al., 2016). However, the cymba concha offers some disadvantages in terms of complexity of electrical stimulation by requiring to insert and/or hold an electrode against the concha, as opposed to having to clip onto the tragus.

Lastly, laterality of aVNS is debated. The stimulation of the left or right aVN cannot be expected to yield different physiological effects since afferent information from both sides are centrally merged in the brainstem (Chen et al., 2015b), and the right and left aVN show comparable counts of Aβ fibers (Safi et al., 2016). This is in clear contrast to the invasive cervical VNS with dominant lateral effects, in which, for instance, the right side stimulation recruits predominantly the sinoatrial node (e.g., with the associated bradycardia) and the left side the atrioventricular node. However, simultaneous activation of the left and right aVN may potentially boost stimulation effects due to increased sensory input to the brainstem.

## Future Directions

Even though clinical effects of aVNS and functional projections of aVN are quite consistent, anatomical projections of auricular nerves still lack clarity (Burger and Verkuil, 2018). This complicates identification of biophysical mechanisms, especially in cardiovascular responses (Mahadi et al., 2019) as well as in nociception, and thus warrants further anatomical or chemical tracing studies.

The experimental and clinical efficiency of different stimulation patterns needs to be investigated, not only on the local auricular level – as accessible with neuronal models – but also on the systemic body level. The relevance of such investigations is indirectly requested by observations that a strong VNS tends to facilitate atrial fibrillation, whereas a moderate VNS tends to inhibit it (Chen et al., 2015a). Likewise, the intermittent VNS attenuated the sympathetic tone but not the continuous VNS (Buchholz et al., 2014), whereas hemodynamic changes were observed only at relatively high VNS intensities (Ness et al., 2000).

Reported data on VNS and aVNS should be interpreted in light of different stimulation methods used in VNS and aVNS and even different methods within aVNS itself (Kaniusas et al., 2019). In fact, aVNS can be operated as the percutaneous stimulation with a quite focused recruitment of local auricular nerves or as the transcutaneous stimulation with a rather diffuse stimulation and thus a potential recruitment of aVN and non-vagal endings. However, it is important to recognize that whether aVN is stimulated, either itself or with/without other auricular nerves, is potentially less important than recognizing that the clinical effects in humans are real. Here Figure 3 illustrates clinical applications and the relevance of the associated scientific reports that substantiates the authenticity of aVNS effects. It should be noted that the used selection of publications is potentially biased since the present review is a descriptive but not a systematic review.

The prediction of responsiveness to aVNS (or VNS) needs further investigations with respect to baseline conditions of patients as well as their gender and age. For instance, baseline conditions in HRV parameters have shown different influence in VNS and aVNS (Clancy et al., 2014; Liu et al., 2017), which potentially indicates different mechanisms of action behind VNS and aVNS. Even in aVNS alone, HRV studies delivered diverging results (De Couck et al., 2016; Antonino et al., 2017).

Further basic research in animals and humans is clearly warranted in order to identify and understand reproducible aVNS biologic effects on the single organ and at the systemic level. The transition of animal aVNS models to humans is hardly addressed and should be investigated. The clinical potential of aVNS in treating different medical conditions should be clarified based on solid and prospective clinical studies in humans, whereas aVNS is typically applied for multimodal disease management. For instance, aVNS effects are rather consistent in chronic but not acute pain (Sator-Katzenschlager and Michalek-Sauberer, 2007). Sustainable physiological effects of aVNS in humans should be quantified.

The paired delivery of VNS and rehabilitative training – known as Paired Vagus Stimulation – indicates improved task-specific plasticity in the brain, providing a more effective rehabilitation. The paired approach was tested in stroke (Dawson et al., 2015), tinnitus (De Ridder et al., 2013), post-traumatic stress disorders (Peña et al., 2012), and other diseases (Hays, 2015). This may indicate potential advantages of aVNS pairing with rehabilitative stimuli that call for future research in this area.

## Conclusion

Auricular vagus nerve stimulation modulates parasympathetic activity of the body inhibiting detrimental sympatho-dominant processes, improves peripheral perfusion due to decreased sympathetic activity, and reduces over-inflammation. Arguable, aVNS has likely a great potential to treat numerous diseases linked with ANS. aVNS may eventually serve as an alternative to reduce opioid use in chronic disease, potentially helping to fight the opioid crisis. Most clinical effects sustain the end of aVNS application, indicating the presence of long-lasting aVNS effects due to inert processes such as the release of neurotransmitters and endorphins, long-term brain plasticity, and self-sustaining changes in the sympathovagal balance.

Auricular vagus nerve stimulation is not close to prime time but gains momentum as a new way of treatment by harnessing the body’s own protective mechanisms beyond the mediation of symptoms, warranting further scientific and clinical research on aVNS. aVNS makes possible to modulate the mind’s great influence over the body via the vagus nerve. An exciting new therapeutic era may begin allowing clinicians to use both electroceuticals and pharmaceuticals in a complementary way for the clear benefit of patients.

## Author Contributions

EK, SK, MT, and JS contributed conception and design of the review. EK wrote the first draft of the manuscript. EK and SK performed the initial literature review. FP and MP contributed to anatomical sections of the manuscript. MT, RG, WK, and GV contributed to biological sections of the manuscript. SK and SL contributed to regulatory section of the manuscript. AC, ET, AMS, TT, and WJ contributed to numerical section of the manuscript. VM and AL contributed to sections on personalized stimulation. NI and AS contributed to engineering sections of the manuscript. AK and BP contributed to clinical sections of the manuscript. All authors contributed to manuscript revision, read and approved the submitted version.

## Conflict of Interest Statement

EK and SL were employed, and SK is employed by company SzeleSTIM GmbH. JS receives honoraria from SzeleSTIM GmbH and owns patents in the field of the auricular vagus nerve stimulation. EK, SK, and JS are shareholders of SzeleSTIM GmbH. The remaining authors declare that the research was conducted in the absence of any commercial or financial relationships that could be construed as a potential conflict of interest.
